# Development and validation of a prognostic computed tomography scoring model for functional outcomes in patients with large hemispheric infarction following decompressive craniectomy

**DOI:** 10.3389/fneur.2024.1336121

**Published:** 2024-01-23

**Authors:** Yutong Zhao, Yuguang Tang, Zongyi Xie

**Affiliations:** Department of Neurosurgery, The Second Affiliated Hospital, Chongqing Medical University, Chongqing, China

**Keywords:** large hemispheric infarction, decompressive Craniectomy, prediction model, functional outcome, cohort study

## Abstract

**Background:**

There is no established prognostic scoring system developed for patients with large hemispheric infarction (LHI) following decompressive craniectomy (DC) based on imaging characteristics. The present study aimed to develop and validate a new computed tomography scoring model to assess the 6-month risk of poor functional outcomes (modified-Rankin scale [mRS] score of 4–6) in patients with LHI receiving DC.

**Methods:**

This retrospective cohort study included patients at two tertiary stroke centers. A prediction model was developed based on a multivariable logistic regression. The final risk factors included the ASPECTS (Alberta Stroke Program Early Computed Tomography Score), longitudinal fissure cistern, Sylvian fissure cistern, and additional vascular territory involvement. 1,000 bootstrap resamples and temporal validation were implemented as validations for the scoring system.

**Results:**

Of the 100 individuals included in the development cohort, 71 had poor functional outcomes. The scoring model presented excellent discrimination and calibration with C-index = 0.87 for the development cohort, and C-index = 0.83 for the temporal validation cohort with non-significant Hosmer-Lemeshow goodness-of-fit test. The scoring model also showed an improved AUC compared to the ASPECTS. For each point in the score model, the adjusted risk of poor functional outcomes increase by 47.8% (OR = 1.48, *p* < 0.001). The scores were inversely correlated with MAP (mean arterial pressure, paired *t*-test, *p* = 0.0015) and CPP (cerebral perfusion pressure, rho = −0.17, *p* = 0.04).

**Conclusion:**

In patients with LHI following DC, the score system is an excellent predictor of poor functional outcomes and is associated with CPP and MAP, which might be worth considering in clinical settings after further external validation.

## Introduction

Up to 10% of all ischemic strokes (IS) are estimated to be large hemispheric infarctions (LHIs), with the most commonly supported definition referring to a severe form of ischemic stroke affecting the majority or entirety of the middle cerebral artery (MCA) distribution areas with or without anterior cerebral artery (ACA) and posterior cerebral artery (PCA) involvement, that is characterized by the development of life-threatening cerebral edema (1–3). Previous studies revealed that a decompressive craniectomy (DC) is a lifesaving treatment that can improve the survival of patients with LHI who experience malignant brain swelling (1, 4). Individuals with LHIs after DC still experienced long hospital stay and high cost; thus, the prediction of postoperative outcomes is of significant importance for the individualized treatment in neurosurgical practice.

Brain computed tomography (CT) is the first choice for examination after DC and provides essential therapeutic information. ASPECT is widely applied in clinical practice to assess the extent of early ischemic changes on brain imaging and to predict the prognosis of individuals with IS (5, 6). Although the ASPECTS estimations were originally based on intraparenchymal hypoattenuation and focal swelling, the quantification of occupying effects (midline shifts, compressed brain cisterns, etc.), which should be non-negligible for LHIs, was not evaluated in detail. In 2005, the Rotterdam CT score, which is composed of the status of basal cisterns, midline shift, and types of mass lesions or intracranial hemorrhage, was developed for traumatic brain injury (TBI) (7). Recent studies have further confirmed the predictive value of the Rotterdam CT score for intracranial hypertension and its prognostic ability in patients undergoing DC following TBI (8, 9). Thus, some characteristics (especially the status of cisterns and midline shift) in the Rotterdam CT score might be important replenishments for the prognosis prediction of LHIs in terms of their reflection of occupying effects and established predictive capability in potentially intracranial hypertension.

We therefore developed and validated a new scoring model combining the characteristics of the ASPECTS and Rotterdam CT score, to determine whether the new model could exhibit a good predictive value for functional outcome in patients with LHI following DC.

## Methods

### Study design and source of data

The study was approved by the local institutional review board (The National Drug Clinical Trial Institution) and the requirement for informed consent was waived.

#### Development cohort

Our development data were obtained from a cohort of two comprehensive tertiary stroke centers affiliated with the same university. Electronic health records were used to systematically collect data during hospitalization, and telephone interviews were used to collect data after discharge. We identified a cohort of patients who were admitted between January 2015 and January 2021. The 6-month functional outcomes after surgery were recorded. Patients were eligible for inclusion in the study if they met the following criteria:

1. Underwent DC due to large hemispheric infarction, as diagnosed by physicians or other specialists.
2. At least 18 years of age;
3. Functionally independent before stroke (modified Rankin Scale score ≤ 2).

Patients were excluded due to the following criteria:

1. LHI after subarachnoid hemorrhage;
2. Extravasation of the contrast medium happening during endovascular therapy (mechanical thrombectomy, etc.) and validated by non-contrast CT scan (Contrast extravasation with corresponding hyperdense area on non-contrast CT might impact the accuracy of data collection on the evaluation of ASPECTS and the status of cisterns);
3. Did not undergo cranial CT within 7 days post-DC;
4. Infarcted brain tissue resection was performed during DC.

#### Temporal validation cohort

Temporal validation included a retrospective cohort from the same 2 comprehensive tertiary stroke centers in which DC was performed between January 2021 and February 2023. The inclusion and exclusion criteria were the same as the development cohort.

### Candidate predictors

**Figure 1:**
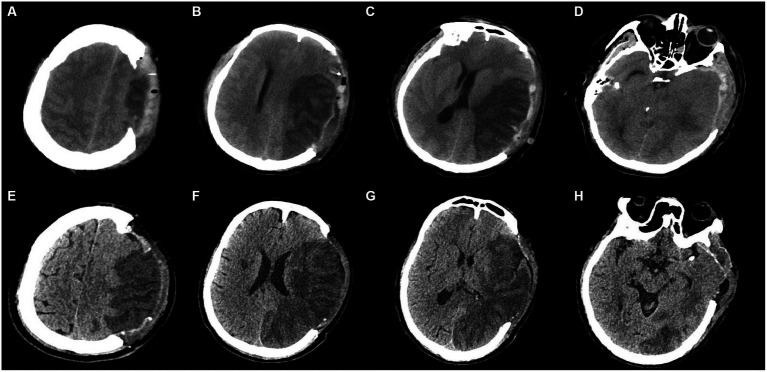
Illustrations of CT characteristics. Subfigures of A-D and E-H were from 2 patients, respectively. **(A–D)**: **(A)** At the vertex: the status of sulcal effacement: *bilateral effacement for this patient*; **(B)** at the body of lateral ventricle (rostral to basal ganglia): any involvement of ACA, and M4-6 in ASPECTS: *involvement of M5, M6 for this patient*; **(C)** at thalamus and basal ganglia: the status of longitudinal fissure in front of corpus callosum, the status of Sylvian fissure, any involvement of PCA, midline shift, and ASPECTS: *bilaterally compressed Sylvian fissure, compressed longitudinal fissure, noninvolvement of PCA, and involvement of M2, M3, insula in ASPECTS for this patient*; **(D)** at midbrain: the status of basal cistern (the status of ambient cisterns in particular): *compressed basal cistern (bilaterally compressed ambient cisterns in particular) for this patient.* **(E–H)**: **(E)** At the vertex: the status of sulcal effacement: *ipsilateral effacement for this patient*; **(F)** at the body of lateral ventricle (rostral to basal ganglia): any involvement of ACA, and M4-6 in ASPECTS: *involvement of M4-6 for this patient*; **(G)** at thalamus and basal ganglia: the status of longitudinal fissure in front of corpus callosum, the status of Sylvian fissure, any involvement of PCA, midline shift, and ASPECTS: *bilaterally present Sylvian fissure, present longitudinal fissure, involvement of PCA, and involvement of M1-3, insula, internal capsule, lentiform in ASPECTS for this patient*; **(H)** at midbrain: the status of basal cistern (the status of ambient cisterns in particular): *present basal cistern (bilaterally present ambient cisterns in particular) for this patient*.

Our candidate predictors focused on non-contrast CT characteristics and were composed of three parts. The first part was the evaluation of ASPECTS (focusing on the ischemia extent) (5). The second part was items yielded from the novel Rotterdam CT Score (focusing on the occupying effect) (9). The status of basal cisterns, midline shift, sulcal effacement, and types of mass lesions or intracranial hemorrhage were preceding reported items in the novel Rotterdam CT Score. Considering the discrepancies between TBI and LHI, we deleted several items from the Rotterdam CT Score (epidural mass lesion and intraventricular blood or subarachnoid hemorrhage) in our candidate predictors. Remaining cistern status, midline shift, and sulcal effacement were all recorded as our candidate predictors. The evaluation of sulcal effacement also accords with the novel Rotterdam CT Score (9). Sulcal effacement was evaluated at the vertex, in the most rostral 2.5 cm of the CT of the head. Partial effacement of the sulci was recorded as present. Notably, besides the status of basal cisterns written up, the status of other important cisterns was recorded as well (longitudinal fissure [present versus compressed], Sylvian fissure [bilaterally present, ipsilaterally compressed, bilaterally compressed], ambient cisterns [bilaterally present, ipsilaterally compressed, bilaterally compressed]). The status of longitudinal fissure was evaluated at the level of thalamus and basal ganglia. Only the longitudinal fissure in front of corpus callosum was taken into consideration. Additionally, the third part of our candidate predictors was the involvement of additional vascular territories (categories: involvement or non-involvement, additional vessels, including the ACA or PCA). The illustrations of these CT characteristics were illustrated in Figure 1. CT characteristics were collected within 7 days post-DC. If a patient underwent CT more than once within 7 days after surgery, all CT studies and corresponding times were recorded. Considering the potential discrepancy in the course of edema (a fulminant course [within 24–36 h], a gradually progressive course [over several days], an initial worsening course followed by a plateau and resolution [approximately a week], or an initial brain swelling course during the first 3–5 days and disappearance after weeks), only the characteristics of the last CT scan (postoperative 4–7 days in our cohort, when most individuals had experienced the peak plateau of brain swelling) were determined as potential variables for predictor selection (2, 3). All candidate predictors were assessed before the outcome assessment.

### Other baseline characteristics

The baseline patient characteristics collected for this study included age, sex, comorbidities, lifestyle, admission variables, interval between infarction and decompression, ASPECTS values before decompression, midline shift before decompression, and brain herniation. Patients with a history of antithrombotic medication within 3 days or with brain herniation before DC were prioritized for implantation of an ICP monitor with an intraparenchymal probe in the ischemic hemisphere at the two centers. MAP and ICP data collected on an hourly basis within 7 days post-DC were also continuously recorded. CPP was calculated based on the difference between the MAP and ICP.

### Surgical procedure

The main surgical procedure was consistent with previous trials of DC in LHI (*DEcompressive Surgery for the Treatment of malignant INfarction of the middle cerebral arterY II [DESTINY II] and the Hemicraniectomy After Middle Cerebral Artery infarction with Life-threatening Edema Trial [HAMLET]*). Procedures were performed by removing a bone flap including portions of the frontal, parietal, and temporal squama, ultimately achieving a craniectomy area with an anterior-to-posterior diameter ≥ 12 cm. The surgical procedures were detailed in Supplementary Digital Content 1.

### Outcome

The primary endpoint of this study was poor functional outcome, which was evaluated using the 6-month mRS score. We chose an mRS score of 0–3 as a favorable functional outcome, which is consistent with previous studies (10–12). The 6-month functional outcomes were evaluated by another experienced mRS certified rater (YGT) who was blinded to the potential predictor. Participants and their caregivers (if any) were interviewed. The follow-up data were collected through telephone interview in September 2023.

### Statistical analysis

#### Missing data

Multiple imputations with chained equations were used to impute missing data. Five imputations were performed. Variables including age, NIHSS score at admission, SBP, and DBP at admission, all post-DC CT characteristics, and functional outcomes were included in the imputation procedure. Rubin’s rules were applied to combine the results across the imputed datasets (13).

#### Model development

Candidate predictors were selected by LASSO regression using the glmnet package in R. The LASSO method determines the optimal number of structures when the mean-square fitting error is at a minimum plus one standard deviation. We added all candidate predictors in LASSO regression. By bootstrapping, we repeated the LASSO regression (the selection) 1,000 times. Only the variables that were selected at least 50% of the time during 1,000 bootstrapping samples would be selected as final predictors. Factors with a relative selection frequency < 50% were excluded. The final prediction model was based on the logistic regression of the final selected predictors. No interaction terms were included in the final model. Diagnostic analyses of the final model included an examination of nonlinear relationships (evaluated by RCS regression), influential points (assessed by Cook’s distance), and multicollinearity (detected by the variance inflation factor of each covariate). A point scoring system (post-decompressive CT score [pDCT-score]) was developed to facilitate clinical application.

#### Grading with the scoring system

A scoring system was then developed using the final prediction model. The presence of a longitudinal fissure cistern and Sylvian fissure cistern, non-involvement of additional vascular territories, and an ASPECTS value of 7 were set as the baseline (reference values). Seven was the highest ASPECTS value in our cohort; therefore, cases with an ASPECTS >7 were not included in our prediction model. The proportional weight for each variable in the scoring system was evaluated by the variable’s β-coefficients from the final logistic regression. A score chart based on the coefficients was created to estimate the outcome probability.

#### Validation and comparison of the scoring system

The scoring system was validated in development, and temporal validation cohorts by assessing the model discrimination and calibration. The dataset from the development cohort was adopted for the assessment of the internal validation by one thousand bootstrap resamples. C-statistics, Brier score, and Hosmer-Lemeshow goodness-of-fit test were used to assess the discrimination and calibration of the model, respectively. Comparisons between the newly developed scoring model and other variates (ASPECTS and some components of the Rotterdam scale) were performed using the area under the receiver operating curve (AUC). We also compared the predicted risks for our final scoring model (pDCT score) with ASPECTS to calculate the net reclassification improvement (NRI) and integrated discrimination improvement (IDI). The threshold risk was evaluated based on the observed incidence of poor outcomes in the cohort.

#### Explorations on the scoring system

The association between pDCT-score (from the last CT scan) and poor functional outcomes was assessed using multivariate logistic regression. The best cutoff value of our pDCT-score for the prediction of poor functional outcome was identified by Receiver operating characteristic (ROC) curve analysis.

Within 7 days post-DC, the pDCT score was repeatedly calculated using the recorded CT characteristics. We determined three types of changing patterns in the scores: (1) increasing (with the latest pDCT score always higher than the previous one), (2) fluctuating (pDCT scores varying with an initial increase followed by a decrease or vice versa), and (3) decreasing (with the latest pDCT score always lower than the previous one) according to the repeatedly calculated pDCT-score. The relationships between the types and functional outcomes were detailed using logistic regression (mRS score of 0–3; 4–6). The association between MAP and variation in the pDCT score was calculated using a paired *t*-test. The relationship between the pDCT score, and CPP was assessed using the Spearman’s rank correlation test.

The R software was used for all analyses. This study adhered to the TRIPOD (Transparent Reporting of a multivariable prediction model for the Individual Prognosis or Diagnosis) statement for reporting.

## Results

### Study population

**Figure 2:**
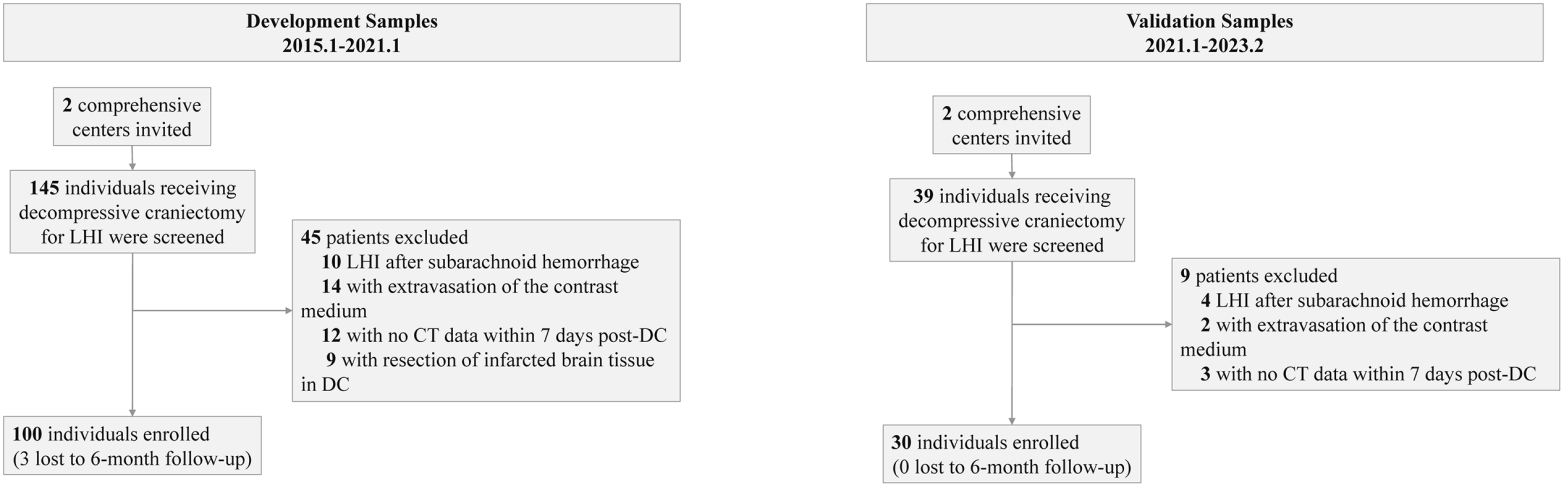
Flowchart showing the study samples.

Before the data were analyzed, multiple imputations were implemented (development cohort: NIHSS score at admission: 2% missing; onset-to-decompression time: 2% missing; 6-month mRS score: 3% missing. Temporal validation cohort: NIHSS score at admission: 1% missing). The final development cohort comprised 100 patients (52 from the first center, 28 from the second center). Of these, 12 had a mRS score of 2, 17 with a mRS score of 3, 28 with a mRS score of 4, 20 with a mRS score of 5, and 23 with a mRS score of 6. The temporal validation cohort composed 30 patients. Among them, 2 had a mRS score of 2, 5 with a mRS score of 3, 9 with a mRS score of 4, 7 with a mRS score of 5, and 7 with a mRS score of 6. The baseline characteristics of the development cohort are presented in Table 1. The comparison between the development cohort and the temporal validation cohort is detailed in Supplementary Digital Content 2. The distribution of important variables between different centers is elaborated in Supplementary Digital Content 3. An overview of the development and validation cohort assembly process is shown in Figure 2.

**Table 1:** Baseline patient characteristics of the development cohort.

Characteristics	Value	mRS of 0–3 *n* = 29	mRS of 4–6 *n* = 71	*p*
Age, years, [mean (SD)]	57 (14)	55 (11)	57 (14)	0.444
Sex [N (%)]				0.342
Male	52 (52.0)	16 (64.0)	36 (50.7)	
Female	48 (48.0)	13 (52.0)	35 (49.3)	
Location [N (%)]				0.287
Left	42 (42.0)	20 (42.5)	22 (41.5)	
Right	58 (58.0)	27 (57.4)	31 (58.5)	
Medical history [N (%)]				
AF	25 (25.0)	1 (3.4)	24 (33.8)	0.003
CHD	8 (8.0)	3 (10.3)	5 (7.0)	0.884
DM	10 (10.0)	6 (20.7)	4 (5.6)	0.056
Hypertension	36 (36.0)	13 (44.8)	23 (32.4)	0.344
Lifestyle [N (%)]				
Smoke	35 (35.0)	12 (41.4)	23 (32.4)	0.533
Drink	29 (29.0)	10 (34.5)	19 (26.8)	0.597
Admission variables				
GCS score, [median (IQR)]	11 [9, 14]	11 [9, 14]	12 [8, 14]	0.527
NIHSS score, [median (IQR)]	16 [13, 18]	13 [11, 17]	17 [14, 18]	0.013
Reperfusion therapies^a^ [N (%)]	42 (42.0)	16 (55.2)	26 (36.6)	0.138
Antithrombotic treatment [N (%)]	55 (55.0)	15 (51.7)	40 (56.3)	0.842
Severity before DC				
Onset to decompression, days, [median (IQR)]	2 [1, 3]	2 [1, 4]	2 [1, 2]	0.060
GCS score before DC, [median (IQR)]	7 [6, 8]	7 [6, 8]	7 [6, 7]	0.245
ASPECTS, [median (IQR)]	1 [0, 3]	3 [1, 4]	0 [0, 3]	<0.001
Midline shift, mm, [median (IQR)]	10.1 [6.9, 12.4]	9.3 [3.9, 11.7]	10.2 [6.9, 12.4]	0.275
Brain herniation, [N (%)]	55 (55.0)	11 (37.9)	44 (62.0)	0.049
BP parameters, mmHg, [mean (SD)]				
Admission SBP	148 (27)	147 (19)	149 (30)	0.710
Admission DBP	88 (14)	94 (14)	86 (14)	0.005
CT characteristics post-DC (4–7 days)				
ASPECTS, [median (IQR)]	0 [0, 3]	3 [1, 4]	0 [0, 2]	<0.001
Additional vascular involvement [N (%)]				0.001
Yes	40 (40.0)	4 (13.8)	36 (50.7)	
No	60 (60.0)	25 (86.2)	35 (49.3)	
Longitudinal fissure [N (%)]				<0.001
Present	50 (50.0)	5 (17.2)	45 (63.4)	
Absent	50 (50.0)	24 (82.8)	26 (36.6)	
Sylvian fissure [N (%)]				<0.001
Present	24 (24.0)	16 (55.2)	8 (11.3)	
Compressed ipsilaterally	49 (49.0)	12 (41.4)	37 (52.1)	
Compressed bilaterally	27 (27.0)	1 (3.4)	26 (36.6)	
Ambient cisterns [N (%)]				0.004
Present	60 (60.0)	24 (82.8)	36 (50.7)	
Compressed ipsilaterally	15 (15.0)	4 (13.8)	11 (15.5)	
Compressed bilaterally	25 (25.0)	1 (3.4)	24 (33.8)	
Sulci^b^ [N (%)]				<0.001
Present	10 (10.0)	8 (27.6)	2 (2.8)	
Effaced ipsilaterally	62 (62.0)	20 (69.0)	42 (59.2)	
Effaced bilaterally	28 (28.0)	1 (3.4)	27 (38.0)	
Midline shift, mm, [median (IQR)]	6.9 [0.0, 11.1]	3.1 [0.0, 7.5]	7.8 [0.0, 12.0]	0.005
ICP monitoring, [N (%)]	48 (48.0)	14 (48.3)	34 (47.9)	1.000
Tracheostomy, [N (%)]	29 (29.0)	11 (37.9)	18 (25.4)	0.310
In-hospital stay, day, [median (IQR)]	25 [11, 42]	29 [25, 47]	20 [18, 30]	0.048
GCS score at discharge, [median (IQR)]	10 [3, 11]	11 [10, 11]	6 [3, 11]	<0.001

a Reperfusion therapies included thrombolysis or endovascular treatment. In detail, 42 individuals accepted reperfusion therapy before DC, and 6 of them experienced thrombolysis only, 21 of them experienced endovascular treatment only, 15 of them received endovascular treatment after thrombolysis.

b Sulcal effacement was evaluated at the vertex, in the most rostral 2.5 cm of the CT of the head. Partial effacement of the sulci was recorded as present.

### Model development

The entire cohort with 1,000 bootstrapping resamples was used to develop the model. The variables involved in the final prediction model included the ASPECTS, longitudinal fissure cistern, Sylvian fissure cistern, and additional vascular territories. The coefficients for each predictor and intercept in the multivariate logistic model are presented in Table 2. For clinical application, we translated the logistic regression model into a score chart, with which the probability of a poor functional outcome (6-month mRS score of 4–6) could be estimated by adding the scores of individual patients (Table 3). The estimate for ASPECTS was converted to 1 as reference value, and other values were converted accordingly. A clean version of the newly development score scale and the relative CT characteristics recording tools were also detailed in Supplementary Digital Content 4. Further examples on the scoring of the new scale were detailed in Supplementary Digital Content 5.

**Table 2:** The coefficients for each predictor and intercept in the multivariate logistic model.

Predictors in CT	Estimate	OR (95% CI)
(Intercept)	−0.49	
Additional vascular involvement	1.65	5.23 (1.31, 20.67)
Longitudinal fissure^a^	0.62	1.86 (1.06, 2.55)
Sylvian fissure		
Ipsilaterally compressed	1.46	4.32 (0.96, 10.79)
Bilaterally compressed	3.21	24.75 (15.91, 29.66)
ASPECTS	−0.33	0.72 (0.51, 0.97)

a Evaluated at the thalamus and basal ganglia level, in front of corpus callosum.

**Table 3:** Post-decompressive CT score, pDCT score.

Predictors in CT			Score
Additional vascular involvement			
No		0	
Yes		5	
Longitudinal fissure^a^			
Present		0	
Compressed		2	
Sylvian fissure			
Bilaterally present		0	
Ipsilaterally compressed		4	
Bilaterally compressed		10	
ASPECTS	0	7	
	1	6	
	2	5	
	3	4	
	4	3	
	5	2	
	6	1	
	7	0	
Sum		24	

a Evaluated at the at thalamus and basal ganglia level, in front of corpus callosum.

The corresponding probabilities are calculated with the formula: Probability (6-m mRS score of 4–6) = 1/ [1+ e^−(−2.84 + 0.33*Sumscore)^].

### Assessment of the scoring model

To assess the performance of our scoring model, we adopted C-statistics to evaluate the model’s discrimination, and Brier scores as well as Hosmer-Lemeshow goodness-of-fit test to calibrate the model. Our model showed excellent discrimination and calibration among the bootstrapping internal validation as well as the temporal validation (Table 4).

**Table 4:** Internal and temporal validation of the scoring model.

	Bootstrapping validation (*n* = 100)	Temporal validation (*n* = 30)
C-statistics (95% CI)	0.87 (0.86, 0.87)	0.83 (0.60, 1.00)
Brier score	0.12	0.11
*p* value of HL goodness-of-fit test	0.45	0.12

HL, Hosmer-Lemeshow.

### Comparison of pDCT score with other variates.

**Figure 3:**
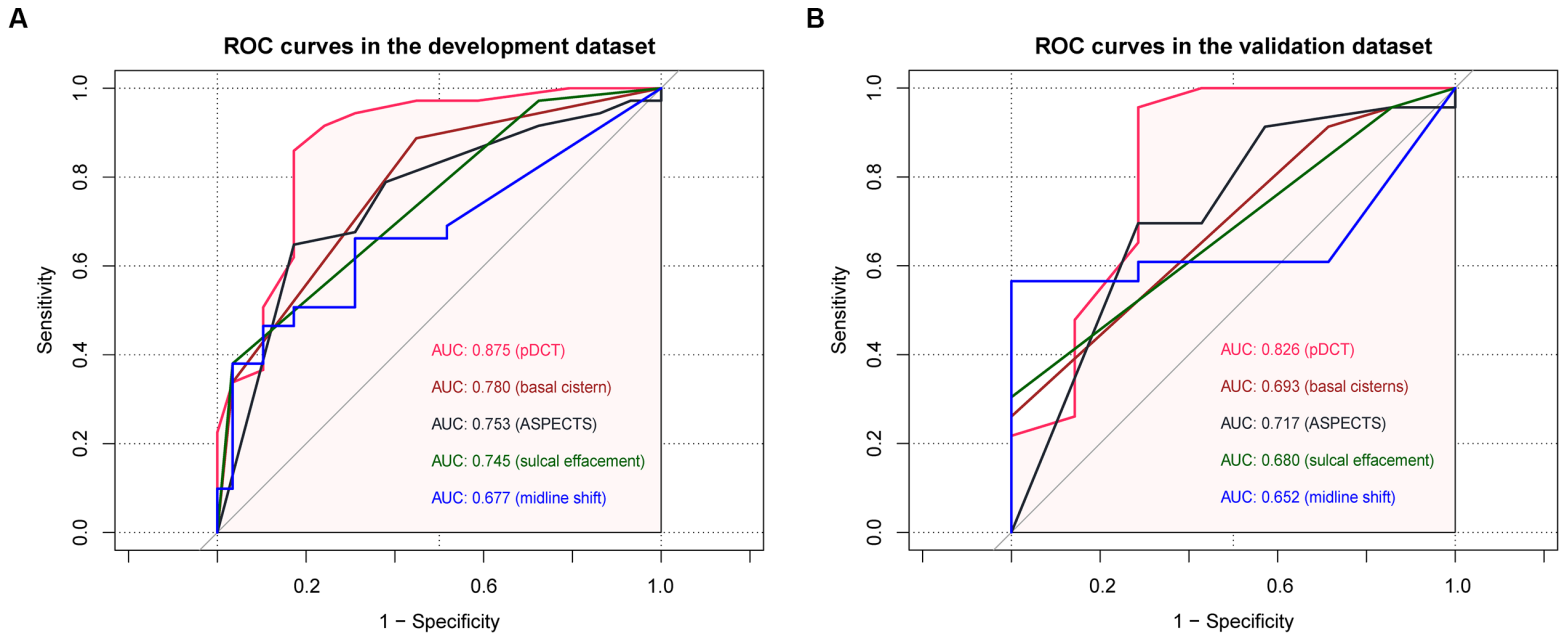
The comparisons between different ROC curves. **(A)** ROC curves in the development dataset. **(B)** ROC curves in the validation dataset.

Comparisons between the newly developed scoring model and other variates (ASPECTS and some components of the Rotterdam scale) were performed. The developed pDCT-score presented a significantly improved AUC (AUC = 0.88, 95%CI = 0.79–0.96) compared to the ASPECTS (AUC = 0.75, 95%CI = 0.65–0.85, *p* value of DeLong test = 0.0003), to midline shift (AUC = 0.68, 95%CI = 0.57–0.78, *p* value of DeLong test <0.001), to the status of basal cisterns (AUC = 0.78, 95%CI = 0.69–0.87, *p* value of DeLong test = 0.008), and to sulcal effacement (AUC = 0.74, 95%CI = 0.66–0.82, *p* value of DeLong test = 0.002) in the development cohort. We classified patients as being at a high risk of poor functional outcome if their 6-month risk was 71% or greater (calculated by the incidence of poor functional outcome in our development cohort). Compared with the ASPECTS model, our pDCT-scoring model showed a categorical NRI of 0.19 (95%CI = 0.01–0.38) with a *p*-value of 0.038, a continuous NRI of 0.84 (95%CI = 0.48–1.20) with a *p*-value of <0.0001, and an IDI of 0.24 (95%CI = 0.16–0.32) with a *p*-value of <0.0001 in the development cohort. In the validation cohort, pDCT-score also performed an improved AUC (AUC = 0.83, 95%CI = 0.6–1.00) compared to the ASPECTS (AUC = 0.72, 95%CI = 0.50–0.94), to midline shift (AUC = 0.65, 95%CI = 0.46–0.84), to the status of basal cisterns (AUC = 0.69, 95%CI = 0.53–0.86), and to sulcal effacement (AUC = 0.68, 95%CI = 0.54–0.82). The comparisons between different ROC curves in the development and validation dataset were delineated in Figure 3.

### Explorations on the scoring system

**Figure 4:**
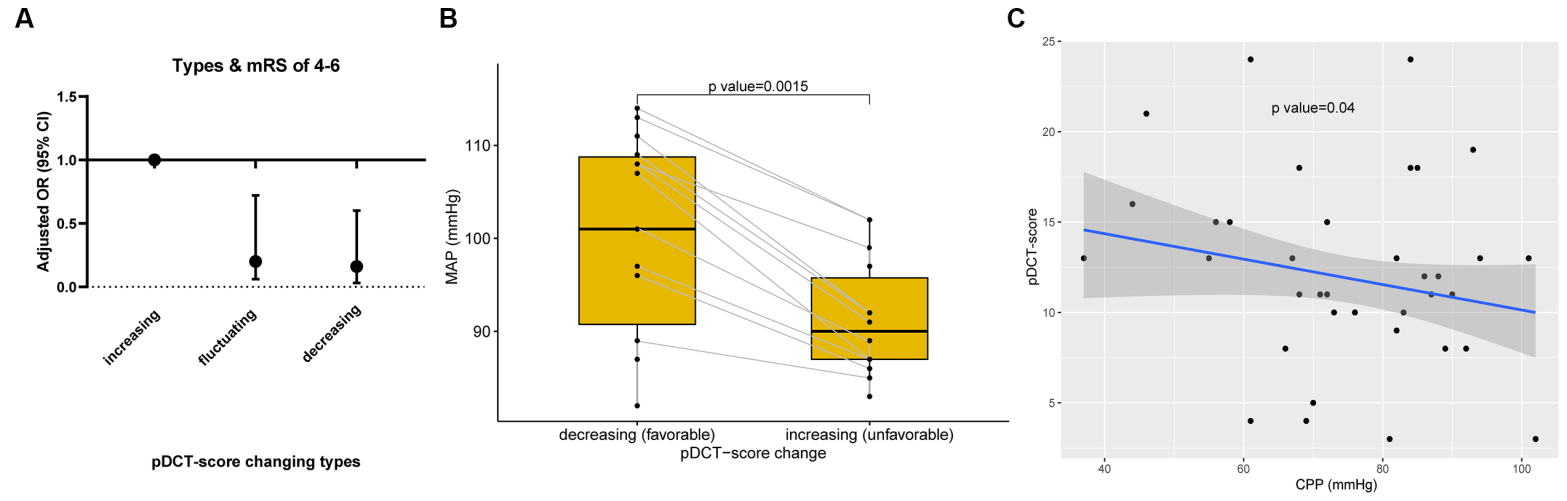
pDCT-score explorations. **(A)** Changing patterns in the pDCT-score and poor functional outcome. Logistic regression (mRS score of 0–3; 4–6) showed that type II (odds ratio [OR] =0.20, 95%CI = 0.06–0.72, *p* = 0.018) and type III (OR = 0.16, 95%CI = 0.03–0.62, *p* = 0.006) were associated with significantly lower risks of poor outcomes than type I (Type I, increasing; Type II, fluctuating; and Type III, decreasing). **(B)** The relationship between MAP and pDCT-score. A paired *t*-test showed a significantly lower MAP during the period with a pDCT score increase than the period with a pDCT score decrease. **(C)** Association between pDCT-score and CPP. Spearman correlation test revealed a significantly negative association between pDCT-score and CPP.

The association between pDCT-score (from the last CT scan) and poor functional outcomes was assessed using multivariate logistic regression. For each point increment in the pDCT-score, the adjusted odds of poor functional outcomes increased by approximately 47.8% (OR = 1.48, 95%CI = 1.26–1.83, *p* < 0.001, adjusted for age, NIHSS score at admission, brain herniation, and smoking). Through ROC analysis, a score of 11 was recognized as the best cutoff value of pDCT-score for the prediction of poor functional outcome with the sensitivity of 0.83, and the specificity of 0.86. We repeatedly calculated the pDCT score within 7 days post-DC using the recorded CT characteristics. Only 4 of 100 patients had 2 CT scans, with the last pDCT scores all higher than their previous one (type I, increasing). All remaining 96 individuals received more than 2 scans during 7 days. There were no patients whose pDCT scores remained unchanged. Three types of changing patterns in the score were determined: Type I, increasing (with the latest pDCT score always higher than the previous one); Type II, fluctuating (pDCT scores varying with an initial increase followed by a decrease or vice versa); and Type III, decreasing (with the latest pDCT score always lower than the previous one). Logistic regression (mRS score of 0–3; 4–6) showed that type II (odds ratio [OR] =0.20, 95%CI = 0.06–0.72, *p* = 0.018) and type III (OR = 0.16, 95%CI = 0.03–0.62, *p* = 0.006) were associated with significantly lower risks of poor outcomes than type I (Figure 4A). For each patient with a type II score-changing pattern, the mean MAP during time periods with increasing and decreasing scores was calculated. The paired *t*-test showed a significantly lower MAP during the period with a raised pDCT-score than the period with a decreased pDCT-score (Figure 4B). Spearman’s correlation test revealed a significantly negative association between the pDCT-score and CPP (rho = −0.17, *p* = 0.04, Figure 4C) after excluding an outlier.

## Discussion

We developed a new scoring model to predict 6-month risk of poor functional outcomes in patients with LHIs following DC. The algorithms incorporated established predictor variables from the Rotterdam CT score and ASPECTS, and new variables associated with the involvement of additional vessels.

The ASPECTS and the involvement of longitudinal and Sylvian fissure cisterns, as well as additional vascular territories, were the main components of our model. The ASPECTS is a recognized indicator inversely associated with the mRS score. A baseline ASPECTS score of seven or less discriminated between independence, dependence, and death at 3-month (5). In our study, the highest observed ASPECTS (post-DC) was seven, which was considered reasonable, as per previous studies. The territory of the ACA or PCA was not considered in the ASPECTS, whereas patients with MCA occlusion were likely to have additional vascular occlusion (3, 4, 14). The occlusion of ACA or PCA was identified as a predictor of in-hospital death caused by brain herniation (OR = 3.3; 95%CI, 1.2–9.4, *p* = 0.02) (15). As such, we included the item “involvement of additional vascular territory” to improve the discrimination of our model. A previous study reported that a compressed basal cistern was associated with raised ICP and CPP < =70 mmHg in TBI (16). Subsequent studies recognized a compressed or absent basal cistern as a valuable predictor of intracranial hypertension and poor prognosis in TBI (7, 9). We expanded the application of this characteristic to LHIs and further separated them into smaller categories which result in the changes of the pDCT-score during the entire disease duration and give value to the analysis of the dynamic variation in scores.

Current theories regarding the course of LHIs vary. A fulminant course (within 24–36 h), a gradually progressive course (over several days), an initial worsening course followed by a plateau and resolution (over approximately a week), or an initial brain swelling course during the first 3–5 days and disappearance after approximately 2 weeks have all been reported (2, 17, 18). Brain swelling of LHI is mainly caused by cytotoxic, ionic, and vasogenic edema, collectively (2, 3). Cytotoxic edema evolves over minutes to hours after the event and declines within 1 day. Ionic edema precedes vasogenic edema by approximately 6 h. The vasogenic edema often peaks at 24–48 h after onset. The median duration from stroke onset to DC was 2 days in our study. In other words, the time of the last CT characteristic we analyzed was 6–9 days from the onset, when most individuals had experienced the three types of edema and been through the peak plateau of brain swelling.

The pathophysiological significance of the pDCT score was also evaluated. An increased pDCT score was associated with higher odds of poor functional outcomes in our analysis. Previous studies have confirmed that decreased MAP values are associated with a greater risk of poor functional outcome (19–22). Given the relationship between MAP and CPP, decreased MAP values ultimately coincided with lower CPP values. With impaired cerebral autoregulation (CA) in LHIs, decreased CPP may contribute to lower CBF and, consequently, a worse functional outcome (23–27). Decreased CBF could appear as an extended ischemia or exacerbated edema on CT, resulting in an increased pDCT-score. The significantly lower MAP during the period with pDCT score increase than the period with pDCT-score decrease in our paired *t*-test (Figure 4B) and the significant inverse relationship between pDCT-score and CPP (Figure 4C) echoed our inference.

Our study has several limitations. First, our model was based on a retrospective observational cohort, and it was difficult to fully rule out potential confounders from unmeasured variables. Second, there was potential bias due to missing data. Third, we explored the potential relationship between our pDCT score and CPP, MAP, whereas we failed to get the data on the pressure reactivity index. Future research could further combine the pDCT score with CPP and the pressure reactivity index, providing more detailed clinical instruction on optimal CPP exploration. Forth, the temporal validation was used in our study, while considering the lower evidence level of temporal validation than external validation, further external validation was needed in the future.

## Conclusion

For patients with LHI after DC, the pDCT-score provided excellent prognostic discrimination and decent calibration. This scoring system is a good predictor of poor functional outcomes and is associated with CPP and MAP. Such an objective, simple, and practical scoring model might be worth considering in clinical settings after further external validation.

## Data availability statement

The raw data supporting the conclusions of this article will be made available by the authors, without undue reservation.

## Ethics statement

The studies involving humans were approved by the National Drug Clinical Trial Institution of the Second Affiliated Hospital of Chongqing Medical University. The studies were conducted in accordance with the local legislation and institutional requirements. The ethics committee/institutional review board waived the requirement of written informed consent for participation from the participants or the participants’ legal guardians/next of kin because it is a retrospective cohort study and no interference was involved.

## Author contributions

YZ: Conceptualization, Data curation, Formal analysis, Investigation, Methodology, Software, Visualization, Writing – original draft. YT: Investigation, Methodology, Validation, Writing – original draft. ZX: Conceptualization, Funding acquisition, Investigation, Project administration, Resources, Supervision, Writing – review & editing.

## Glossary

ACA: anterior cerebral artery
ASPECTS: Alberta Stroke Program Early Computed Tomography Score
AUC: area under the receiver operating curve
CA: cerebral autoregulation
CBF: cerebral blood flow
CI: confidence interval
CPP: cerebral perfusion pressure
CT: computed tomography
DC: decompressive craniectomy
ICP: intracranial pressure
IDI: integrated discrimination improvement
IQR: interquartile range (median)
IS: ischemic strokes
LHI: large hemispheric infarctions
MAP: mean arterial pressure
MCA: middle cerebral artery
mRS: modified-Rankin Scale
NRI: net reclassification improvement
OR: odds ratio
PCA: posterior cerebral artery
pDCT: post-decompressive computed tomography
RCS: restricted cubic spline
TBI: traumatic brain injury
