# Supplementary material for: Development and validation of a prognostic computed tomography scoring model for functional outcomes in patients with large hemispheric infarction following decompressive craniectomy
Source: Front Neurol. 2024 Jan 23;15:1336121. doi: 10.3389/fneur.2024.1336121 (PMC10848326; doi:10.3389/fneur.2024.1336121)
Supplement: Supplementary file 1 [file Data_Sheet_1.docx]

**Supplemental Digital Content 1. Supplemental Methods.** Surgical procedure.

**Supplemental Digital Content 2**. **Supplemental Results.** Comparison between the development cohort and the temporal validation cohort

**Supplemental Digital Content 3**. **Supplemental Results.** The distribution of variables between different centers

**Supplemental Digital Content 4**. **Supplemental Results.** CT characteristics recording tools for pDCT-score

**Supplemental Digital Content 5. Supplemental Results.** Examples on the scoring of the new scale.

**Supplemental Digital Content 1. Supplemental Methods.** Surgical procedure.

Surgical supratentorial decompression was performed by removing a bone flap including portions of the frontal, parietal, and temporal squama, ultimately achieving a craniectomy area with an anterior-to-posterior diameter ≥ 12 cm. When appropriate, additional temporal bone was removed to enable effective exploration of the middle cerebral fossa floor. Intracranial (ICP) monitoring was performed as appropriate by inserting the probe into the brain parenchyma. After opening the dura, an expanded duraplasty was performed. Temporalis resection was performed in cases where bony and dural decompression were insufficient. Infarcted brain tissue was not resected. Although the above procedure was not previously protocolized, it differed little between the centers and could be confirmed by the surgical records for each patient.

**Supplemental Digital Content 2**. **Supplemental Results.** Comparison between the development cohort and the temporal validation cohort

| Characteristics | Development cohort  (n=100) | Validation cohort  (n=30) |
| --- | --- | --- |
| Age, years, (mean (SD)) | 57 (14) | 58 (16) |
| Sex (N (%)) |  |  |
| Male | 52 (52.0) | 20 (67.7) |
| Female | 48 (48.0) | 10 (33.3) |
| Location (N (%)) |  |  |
| Left | 42 (42.0) | 13 (43.3) |
| Right | 58 (58.0) | 17 (56.7) |
| Medical history (N (%)) |  |  |
| AF | 25 (25.0) | 10 (33.3) |
| CHD | 8 (8.0) | 3 (10.0) |
| DM | 10 (10.0) | 2 (6.7) |
| Hypertension | 36 (36.0) | 12 (40.0) |
| Lifestyle (N (%)) |  |  |
| Smoke | 35 (35.0) | 14 (46.7) |
| Drink | 29 (29.0) | 13 (43.3) |
| Admission variables |  |  |
| GCS score, (median (IQR)) | 11 [9, 14] | 13 [10, 14] |
| NIHSS score, (median (IQR)) | 16 [13, 18] | 14 [12, 17] |
| Antithrombotic treatment (N (%)) | 55 (55.0) | 14 (46.7) |
| Severity before DC |  |  |
| Onset to decompression, days, (median (IQR)) | 2 [1, 3] | 2 [1, 3] |
| GCS score before DC, (median (IQR)) | 7 [6, 8] | 7 [6, 8] |
| ASPECTS, (median (IQR)) | 1 [0, 3] | 0 [0, 2] |
| Midline shift, mm, (median (IQR)) | 10.1 [6.9, 12.4] | 10.2 [7.3, 12.8] |
| Brain herniation, (N (%)) | 55 (55.0) | 20 (66.7) |
| BP parameters, mmHg, (mean (SD)) |  |  |
| Admission SBP | 148 (27) | 146 (28) |
| Admission DBP | 88 (14) | 87 (15) |
| CT characteristics post-DC (4-7 days) |  |  |
| ASPECTS, (median (IQR)) | 0 [0, 3] | 0 [0, 2] |
| Additional vascular involvement (N (%)) |  |  |
| Yes | 40 (40.0) | 13 (43.3) |
| No | 60 (60.0) | 17 (56.7) |
| longitudinal fissure cistern (N (%)) |  |  |
| present | 50 (50.0) | 13 (43.3) |
| absent | 50 (50.0) | 17 (56.7) |
| sylvian fissure cistern (N (%)) |  |  |
| present | 24 (24.0) | 4 (13.3) |
| compressed unilaterally | 49 (49.0) | 20 (66.7) |
| compressed bilaterally | 27 (27.0) | 6 (20.0) |
| ambient cisterns (N (%)) |  |  |
| present | 60 (60.0) | 18 (60.0) |
| compressed unilaterally | 15 (15.0) | 6 (20.0) |
| compressed bilaterally | 25 (25.0) | 6 (20.0) |
| Sulci (N (%)) |  |  |
| present | 10 (10.0) | 2 (6.7) |
| compressed unilaterally | 62 (62.0) | 21 (70.0) |
| compressed bilaterally | 28 (28.0) | 7 (23.3) |
| Midline shift, mm, (median (IQR)) | 6.9 [0.0, 11.1] | 7.3 [0.0, 12.5] |
| ICP monitoring, (N (%)) | 48 (48.0) | 19 (63.3) |
| Tracheostomy, (N (%)) | 29 (29.0) | 13 (43.3) |
| In-hospital stay, day, (median (IQR)) | 25 [11, 42] | 26 [9, 40] |
| GCS score at discharge, (median (IQR)) | 10 [3, 11] | 7 [3, 11] |
| Functional outcome, (N (%)) |  |  |
| 6m-mRS score of 0-3 | 29 (29.0) | 7 (23.3) |
| 6m-mRS score of 4-6 | 71 (71.0) | 23 (76.7) |

**Supplemental Digital Content 3**. **Supplemental Results.** The distribution of variables between different centers (development cohort)

| Characteristics | Center 1  (n=52) | Center 2  (n=48) | P value |
| --- | --- | --- | --- |
| Age, years, (mean (SD)) | 57 (14) | 57 (13) | 0.990 |
| Sex (N (%)) |  |  | 0.749 |
| Male | 33 (63.5) | 28 (58.3) |  |
| Female | 19 (36.5) | 20 (41.7) |  |
| Admission variables |  |  |  |
| GCS score, (median (IQR)) | 12 [8, 14] | 11 [10, 14] | 0.969 |
| NIHSS score, (median (IQR)) | 16 [13, 19] | 14 [13, 18] | 0.115 |
| Severity before DC |  |  |  |
| Onset to decompression, days, (median (IQR)) | 2 [1, 2] | 2 [1, 3] | 0.232 |
| GCS score before DC, (median (IQR)) | 7 [6, 8] | 7 [6, 8] | 0.630 |
| ASPECTS, (median (IQR)) | 1 [0, 3] | 1 [0, 3] | 0.817 |
| Midline shift, mm, (median (IQR)) | 10.3 [6.8, 12.3] | 9.9 [6.9, 12.6] | 0.691 |
| Brain herniation, (N (%)) | 27 (51.9) | 28 (58.3) | 0.658 |
| CT characteristics post-DC (4-7 days) |  |  |  |
| ASPECTS, (median (IQR)) | 0 [0, 3] | 1 [0, 3] | 0.402 |
| Additional vascular involvement (N (%)) |  |  | 0.775 |
| Yes | 22 (42.3) | 18 (37.5) |  |
| No | 30 (57.7) | 30 (62.5) |  |
| longitudinal fissure cistern (N (%)) |  |  | 0.841 |
| present | 25 (48.1) | 25 (52.1) |  |
| absent | 27 (51.9) | 23 (47.9) |  |
| sylvian fissure cistern (N (%)) |  |  | 0.134 |
| present | 13 (25.0) | 11 (22.9) |  |
| compressed unilaterally | 21 (40.4) | 28 (58.3) |  |
| compressed bilaterally | 18 (34.6) | 7 (14.6) |  |
| ambient cisterns (N (%)) |  |  | 0.019 |
| present | 30 (57.7) | 30 (62.5) |  |
| compressed unilaterally | 4 (7.7) | 11 (22.9) |  |
| compressed bilaterally | 18 (34.6) | 7 (14.6) |  |
| Sulci (N (%)) |  |  | 0.050 |
| present | 4 (7.7) | 6 (12.5) |  |
| compressed unilaterally | 28 (53.8) | 34 (70.8) |  |
| compressed bilaterally | 20 (38.5) | 8 (16.7) |  |
| Midline shift, mm, (median (IQR)) | 6.8 [0.0, 11.7] | 7 [0.0, 10.1] | 0.896 |

**Supplemental Digital Content 4**. **Supplemental Results.** CT characteristics recording tools for pDCT-score.

| **Level: Rostral to basal ganglia** | | | | | | |
| --- | --- | --- | --- | --- | --- | --- |
| ACA involvement | □ Yes | | | □ No | | |
| ASPECTS | □ M4 | | □ M5 | | □ M6 | |
| **Level: At thalamus and basal ganglia** | | | | | | |
| ASPECTS | □ M1 | | □ M2 | | □ M3 | |
|  | □ C | □ IC | | □ L | | □ I |
| Longitudinal fissure | (in front of corpus callosum) | | | | | |
|  | □ Present | | | □ Compressed | | |
| Sylvian fissure | □ Bilaterally present  □ Ipsilaterally compressed  □ Bilaterally compressed | | | | | |
| PCA involvement | □ Yes | | | □ No | | |

**Supplemental Digital Content 5. Supplemental Results.** Examples on the scoring of the new scale.


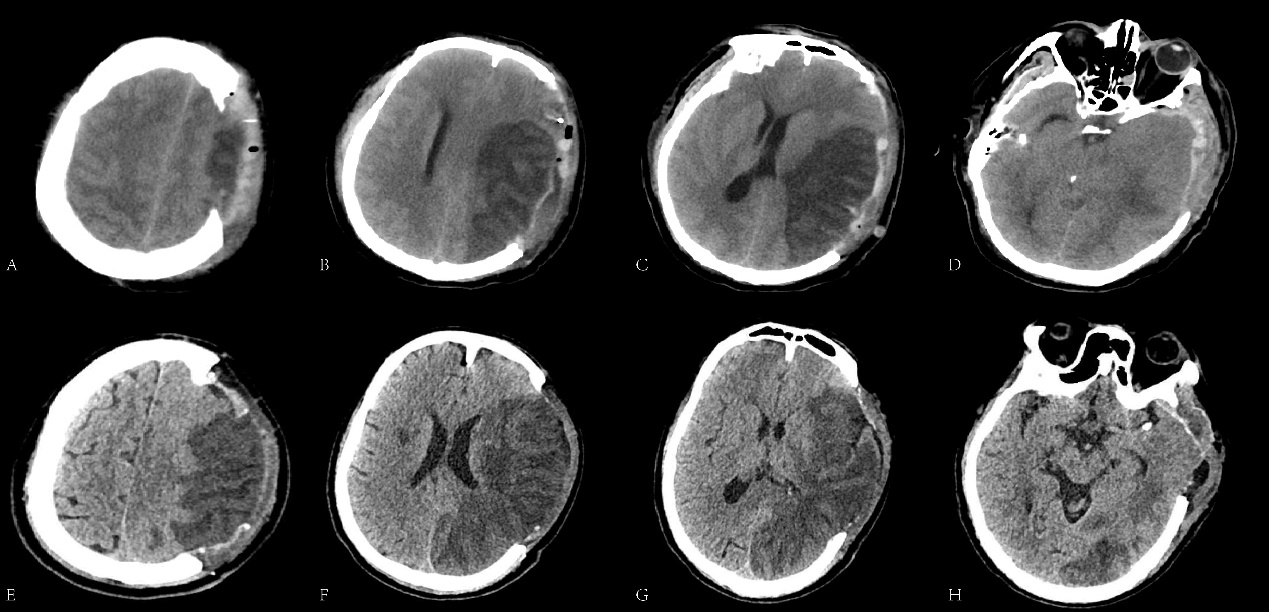


Example 1. (CT images from **Figure 1. A-D**)

CT characteristics recording tools for pDCT-score

| **Level: Rostral to basal ganglia** | | | | | | |
| --- | --- | --- | --- | --- | --- | --- |
| ACA involvement | □ Yes | | | ☑ No | | |
| ASPECTS | □ M4 | | ☑ M5 | | ☑ M6 | |
| **Level: At thalamus and basal ganglia** | | | | | | |
| ASPECTS | □ M1 | | ☑ M2 | | ☑ M3 | |
|  | □ C | □ IC | | □ L | | ☑ I |
| Longitudinal fissure | (in front of corpus callosum) | | | | | |
|  | □ Present | | | ☑ Compressed | | |
| Sylvian fissure | □ Bilaterally present  □ Ipsilaterally compressed  ☑ Bilaterally compressed | | | | | |
| PCA involvement | □ Yes | | | ☑ No | | |

post-decompressive CT score, pDCT score

| **Predictors in CT** | |  | **Score** |
| --- | --- | --- | --- |
| Additional vascular involvement | |  | 0 |
| No | | 0 |  |
| Yes | | 5 |  |
| Longitudinal fissure^ǂ^ | |  | 2 |
| present | | 0 |  |
| compressed | | 2 |  |
| Sylvian fissure | |  | 10 |
| bilaterally present | | 0 |  |
| ipsilaterally compressed | | 4 |  |
| bilaterally compressed | | 10 |  |
| ASPECTS | 0 | 7 |  |
|  | 1 | 6 |  |
|  | 2 | 5 |  |
|  | 3 | 4 | 2 |
|  | 4 | 3 |  |
|  | 5 | 2 |  |
|  | 6 | 1 |  |
|  | 7 | 0 |  |
| Sum | | 24 | 14 |

ǂ: evaluated at the at thalamus and basal ganglia level, in front of corpus callosum

The corresponding probabilities are calculated with the formula:

Probability (6-m mRS score of 4-6) = 1/ [1+ e ^- (-2.84+0.33*Sumscore)^]=85.6%

(The actual 6-m mRS score of this patient was 4.)

Example 2. (CT images from **Figure 1. E-H**)

CT characteristics recording tools for pDCT-score

| **Level: Rostral to basal ganglia** | | | | | | |
| --- | --- | --- | --- | --- | --- | --- |
| ACA involvement | □ Yes | | | ☑ No | | |
| ASPECTS | ☑ M4 | | ☑ M5 | | ☑ M6 | |
| **Level: At thalamus and basal ganglia** | | | | | | |
| ASPECTS | ☑ M1 | | ☑ M2 | | ☑ M3 | |
|  | □ C | ☑ IC | | ☑ L | | ☑ I |
| Longitudinal fissure | (in front of corpus callosum) | | | | | |
|  | ☑ Present | | | □ Compressed | | |
| Sylvian fissure | ☑ Bilaterally present  □ Ipsilaterally compressed  □ Bilaterally compressed | | | | | |
| PCA involvement | ☑ Yes | | | □ No | | |

post-decompressive CT score, pDCT score

| **Predictors in CT** | |  | **Score** |
| --- | --- | --- | --- |
| Additional vascular involvement | |  | 5 |
| No | | 0 |  |
| Yes | | 5 |  |
| Longitudinal fissure^ǂ^ | |  | 0 |
| present | | 0 |  |
| compressed | | 2 |  |
| Sylvian fissure | |  | 0 |
| bilaterally present | | 0 |  |
| ipsilaterally compressed | | 4 |  |
| bilaterally compressed | | 10 |  |
| ASPECTS | 0 | 7 |  |
|  | 1 | 6 |  |
|  | 2 | 5 |  |
|  | 3 | 4 | 6 |
|  | 4 | 3 |  |
|  | 5 | 2 |  |
|  | 6 | 1 |  |
|  | 7 | 0 |  |
| Sum | | 24 | 11 |

ǂ: evaluated at the at thalamus and basal ganglia level, in front of corpus callosum

The corresponding probabilities are calculated with the formula:

Probability (6-m mRS score of 4-6) = 1/ [1+ e ^- (-2.84+0.33*Sumscore)^]=68.8%

(The actual 6-m mRS score of this patient was 4.)
